# Nanobiosensor Based on Sugar Code-AuNPs Aggregation: A Key to Opening New Gates in Rapid Diagnosis of Streptococcal Pharyngitis

**DOI:** 10.3389/fbioe.2022.957271

**Published:** 2022-07-22

**Authors:** Sahar Mohajeri, Saeed Moayedi, Leila Azimi, Mohammad Akrami, Mazda Rad-Malekshahi, Mohammad Reza Fazeli, Fatemeh Fallah, Ismaeil Haririan

**Affiliations:** ^1^ Department of Pharmaceutical Biomaterials, Faculty of Pharmacy, Tehran University of Medical Sciences, Tehran, Iran; ^2^ Pediatric Infections Research Center, Research Institute for Children’s Health, Shahid Beheshti University of Medical Sciences, Tehran, Iran; ^3^ Department of Drug and Food Control, Faculty of Pharmacy, Tehran University of Medical Sciences, Tehran, Iran

**Keywords:** nanobiosensor, gold nanoparticles, streptococcal pharyngitis, gas, diagnosis

## Abstract

Streptococcal pharyngitis is mainly caused by *Streptococcus pyogenes* (GAS), which if left untreated can lead to rheumatic heart disease. The accurate diagnosis of streptococcal pharyngitis is a challenge for clinicians because several symptoms of streptococcal pharyngitis are similar to viral pharyngitis. There are some commercially available biosensors for the rapid diagnosis of streptococcal pharyngitis. Nevertheless, they are not widely used by physicians, mainly because of their high price and dependence on the instrument. Serotype M1 GAS is the most prevalent cause of streptococcal pharyngitis and binds to H-1 antigen, a sugar code found on oral epithelial cells. Here, we present a nanobiosensor based on aggregation of H-1 antigen-conjugated gold nanoparticles for the rapid, qualitative, and quantitative detection of M1 GAS, which is inspired by the sugar code-lectin interaction. It is noteworthy that M1 GAS was detected in a wide concentration range (1 × 10^3^–1×10^6^ CFU/ml) with a linear response and a short detection time of 20 min. Good reproducibility, easy-to-use, and relatively low production cost are among other attractive features of this nanobiosensor. This work provides a strategic roadmap for developing a new generation of biosensors via targeting the sugar code-lectin interaction in future studies.

## Introduction

*Streptococcus pyogenes* (GAS) is a Gram positive bacterium that causes a wide variety of clinical conditions, ranging from acute pharyngitis to severe invasive diseases (Cole et al., 2011). The prevalence of streptococcal pharyngitis is approximately 5%–15% in adults and 20%–30% in children (Frost et al., 2019; Luo et al., 2019). An autoimmune response to streptococcal pharyngitis causes acute rheumatic fever. Cardiac involvement during acute rheumatic fever can lead to rheumatic heart disease (Cunningham, 2012).

A common method for the diagnosis of streptococcal pharyngitis is a physical examination by a physician and then a bacterial culture of throat swab. The accurate diagnosis of streptococcal pharyngitis is difficult for physicians since some symptoms of both streptococcal and viral pharyngitis overlap. Furthermore, the misdiagnosis of streptococcal pharyngitis by clinicians can lead to the unnecessary prescription of antibiotics, which increases the risk of antibiotic resistance. On the other hand, the bacterial culture of throat swab is the gold standard for the diagnosis of streptococcal pharyngitis. However, this method has some limitations like the need for an experienced microbiologist and 1–2 days delay in obtaining results, which may cause a delay in the treatment or unnecessary use of antibiotics. Hence, developing rapid detection tests can help in the early and accurate diagnosis of streptococcal pharyngitis (Luo et al., 2019; Dubois et al., 2021).

Until now, various types of biosensors were developed to diagnose streptococcal pharyngitis, which only some of them were approved by the U.S. FDA for clinical use. Sofia, BD Veritor, and ID NOW are among the most famous commercially available products (US FDA approved)**.** They are rapid tests for the qualitative detection of GAS that are instrument-dependent. Sofia and BD Veritor are based on antibody-antigen interaction, and ID NOW is based on isothermal nucleic acid amplification technology. Owing to their high specificity and sensitivity, these products can help in the accurate diagnosis of streptococcal pharyngitis (Bulut et al., 2020; Thompson and McMullen, 2020; Quidel Corporation 2015; U.S. Food and Drug Administration 2013; U.S. Food and Drug Administration 2018; Abbott Diagnostics Scarborough 2020). Nevertheless, they are not widely used by clinicians worldwide, which may be in terms of their high price and dependence on the instrument.

If a biosensor is developed that not only aids in the quick and qualitative detection of GAS without using a specialized instrument, but also has a cheap manufacturing cost and the capacity to do quantitative analysis, it might be a game-changer in the diagnosis of streptococcal pharyngitis. It is important to note that M1 GAS is among the most frequently isolated serotypes from streptococcal pharyngitis. M1 protein (a main virulence factor of M1 GAS) binds to H-1 antigen, a sugar code present on oral epithelial cells. M1 protein is regarded as a type of lectin (Cole et al., 2011; De Oliveira et al., 2017; Poole et al., 2018). Several cellular functions ranging from recognizing the pathogen to cell adhesion are mediated through lectins. They distinguish carbohydrates bound to lipids and proteins within the extracellular matrix and on the cell surface. These carbohydrates are considered as sugar codes (Vasta, 2009).

On the other hand, gold nanoparticles (AuNPs) exhibit the intense absorption peak around 520 nm in terms of so-called surface plasmon resonance (SPR). AuNPs are of particular interest for broad use in medicine and biology, mainly because of their ease of synthesis, chemical inertness, and tunable physical properties (De et al., 2008; Yan et al., 2012). The nanobiosensors based on the aggregation of AuNPs have recently found a lot of diagnostic applications especially in the diagnosis of pathogens, where the presence of a specific antigen in a colloidal medium containing bioreceptor–AuNPs conjugates results in the aggregation of nanoparticles (Cao et al., 2009; Liu et al., 2009; Chi et al., 2010; Wang et al., 2010; Mou et al., 2019; Pramanik et al., 2021). This study aimed to detect M1 GAS using a novel nanobiosensor. In this study, we developed a relatively affordable nanobiosensor based on the aggregation of H-1 antigen-conjugated AuNPs for the rapid, qualitative, and quantitative detection of M1 GAS. Due to easy-to-use for qualitative detection without reliance on the specialized equipment, and relatively low production cost, H-1 antigen-conjugated AuNPs can be considered a novel nanobiosensor for the detection of M1 GAS that may result in a strategic breakthrough in the diagnosis of streptococcal pharyngitis.

## Materials and Methods

### Materials and Instruments

Tetrachloroauric(III) acid trihydrate (HAuCl₄.3H₂O, 99.5%), trisodium citrate (C₆H₅Na₃O₇, 99.0%), avidin from egg white, and biotin-4-fluorescein were purchased from Sigma-Aldrich (Germany). Lacto-N-fucopentaose I-biotin (H-1-biotin) was purchased from Dextra Laboratories (United Kingdom). *Streptococcus pyogenes* M1 (ATCC^®^ 700294), *Streptococcus pyogenes* M6 (ATCC^®^ BAA-946), *Streptococcus pyogenes* M12 (ATCC^®^ BAA-1315), and *E. coli* O157:H7 (ATCC35150) were purchased from ATCC (United States). UV–Vis absorption spectrophotometer (CE7500, United States), Transmission Electron Microscope (Zeiss EM 900, Germany), Dynamic Light Scattering (Scatteroscope I, South Korea), and fluorescence spectrometer (PerkinElmer, United States) were used.

### Synthesis of AuNPs

AuNPs were synthesized by reduction of HAuCl₄ with trisodium citrate (Turkevich et al., 1951). Briefly, 250 μl of 140 mM solution of trisodium citrate was rapidly added to 50 ml of 0.25 mM solution of HAuCl₄ under vigorous stirring and reflux conditions. After 10 min, the solution was slowly cooled down to room temperature. Characterization of AuNPs was carried out using UV–Vis absorption spectroscopy, Transmission Electron Microscope (TEM), and Dynamic Light Scattering (DLS).

### Preparation of Avidin-Conjugated AuNPs

Avidin–biotin interaction was used to bind AuNPs to biotinylated H-1 antigen. Avidin, a glycoprotein from egg white, has a positive charge at pH below its isoelectric point (about 10) and citrate-reduced colloidal AuNPs have a negative charge. AuNPs were electrostatically bound to avidin (Petronzelli et al., 2010; Montenegro et al., 2013; Ahmed et al., 2014; Bajpayee et al., 2016; Jain and Cheng, 2017). The pH of synthesized colloidal AuNPs was in the range of 4–5. The pH of colloidal AuNPs was set to 6–7 by adding NaOH solution. In the next step, 100 μl of avidin with concentrations of 50, 75, 100, 125, and 150 μg/ml was added individually to 900 μl of colloidal AuNPs. All samples were incubated for 1 h at 25°C, and then were centrifuged at 8,500 rpm for 20 min and the supernatant containing unbound avidin was removed. Assessment of AuNPs’ binding to the avidin was performed using Zigmondy’s test (Slot and Geuze, 2007; Ríos-Corripio et al., 2013). 80 μl of NaCl solution of 10% w/v was added to 1 ml of avidin-conjugated AuNPs (avidin-AuNPs) prepared in previous step. Photography and UV–Vis absorption spectroscopy were applied to determine the best conjugation condition. All experiments were carried out in triplicates.

### Preparation of H-1 Antigen-Conjugated AuNPs

100 μl of H-1-biotin with concentrations of 10, 20, 30, and 40 μg/ml was individually added to 900 μl of avidin-AuNPs. All samples were incubated for 1 h at 25°C and then centrifuged at 8,500 rpm for 20 min and the supernatant containing unbound H-1-biotin was removed. Biotin-4-fluorescein was applied to evaluate the avidin-AuNPs’ binding to the H-1-biotin. Biotin-4-fluorescein is used to detect biotin-binding sites by fluorescence spectroscopy (McMahon, 2008; Waner and Mascotti, 2008; Chivers et al., 2010; Mittal and Bruchez, 2011; Sun et al., 2012). 100 μl of biotin-4-fluorescein with concentration of 50 μg/ml was individually added to 900 μl of H-1 antigen-conjugated AuNPs (H-1-AuNPs). All samples were incubated for 1 h at 25°C, and then were centrifuged at 8,500 rpm for 20 min and the supernatant containing unbound biotin-4-fluorescein was removed. Assessment of avidin-AuNPs’ binding to the H-1-biotin was performed using fluorescence spectroscopy (λex 494 nm; λem 522 nm) on supernatant to determine the best conjugation condition. All experiments were carried out in triplicates.

### Detection Assay of M1 GAS

To biomimic natural saliva medium, a saliva sample was collected after a physical examination of a healthy individual by the physician. To ensure no bacterial contamination of this sample with GAS, microbial evaluation was performed by Pouya Gene Azma Company (Tehran, Iran). This saliva sample was used as a medium for the detection assay of M1 GAS. In the next step, M1 GAS was recovered from the stocks on Blood agar with 5% sheep blood and then the plates were incubated under 5% CO_2_ at 37°C for 1 day. Colonies were counted via the OD measurement at 600 nm after 1 day of growth. *Streptococcus pyogenes* M6 (M6 GAS), *Streptococcus pyogenes* M12 (M12 GAS), and *E. coli* O157:H7 were considered as controls. M6 GAS and M12 GAS were treated at the same mentioned conditions. (Camara et al., 2013; Parvizi et al., 2014; Kebede et al., 2021). *E. coli* O157:H7 was recovered from the stocks on Nutrient Broth and then the plates were incubated at 37°C for 1 day. Colonies were counted through the OD measurement at 600 nm after 1 day of growth (Sağdıç et al., 2002; Banerjee et al., 2016; Li et al., 2017).

Then, saliva samples infected with M1 GAS, M6 GAS, M12 GAS, and *E. coli* O157:H7 at concentrations of 1 × 10^2^, 1 × 10^3^, 1 × 10^4^, 1 × 10^5^, 1 × 10^6^, and 1 × 10^7^ CFU/ml were prepared and 100 μl of samples were added individually to 900 μl of H-1-AuNPs. All samples were incubated for 20 min at 25°C. The samples were assessed using photography and UV–Vis absorption spectroscopy. All experiments were carried out in triplicates.

## Results and Discussion

### Characterization of AuNPs

**FIGURE 1:**
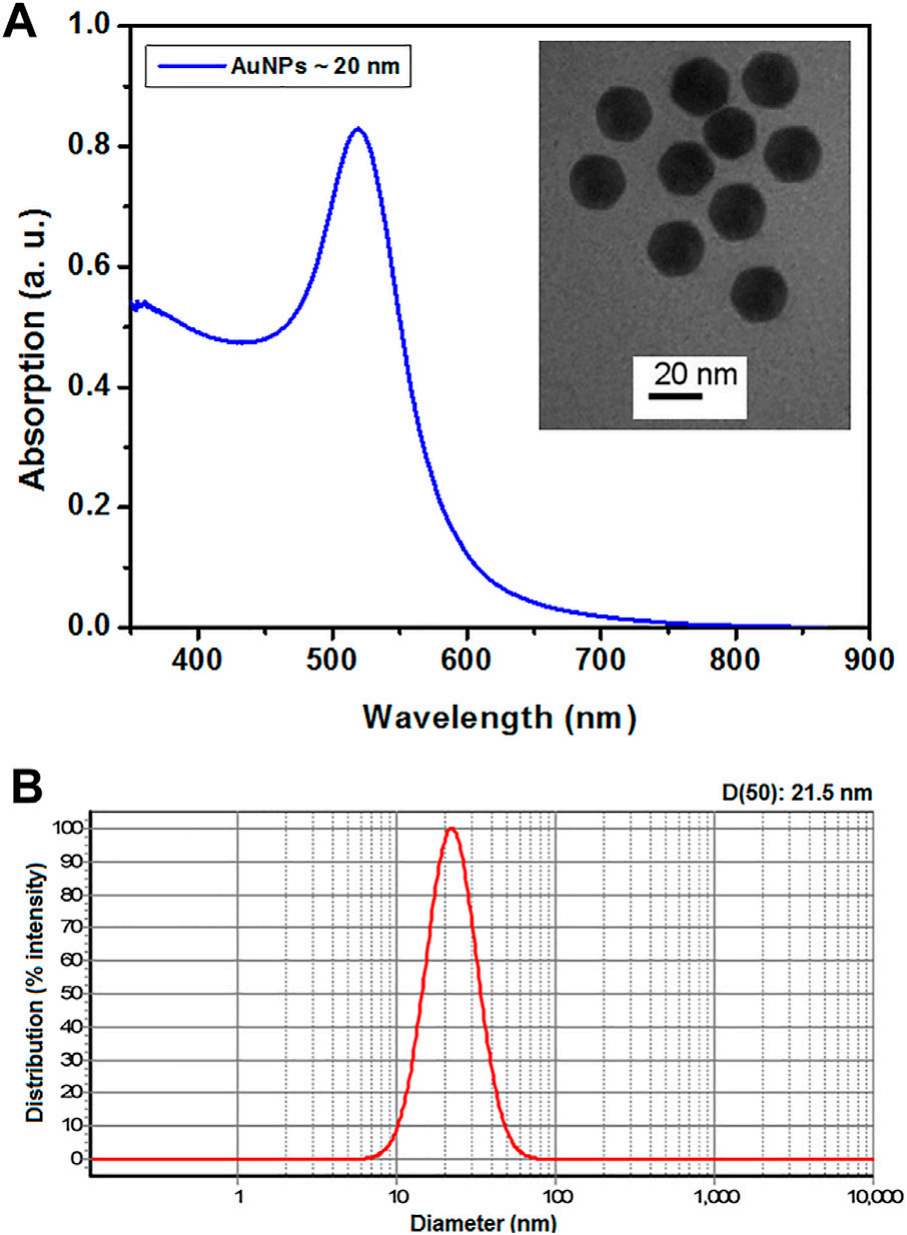
UV–Vis absorption spectrum of AuNPs and transmission electron micrograph of the same (inset) **(A)**. The particle size distribution of AuNPs measured by DLS **(B)**.

UV–visible absorption spectrum and transmission electron micrograph of AuNPs are presented in Figure 1A. Based on this spectrum and the size estimation method presented by Haiss et al. (2007), the average size of AuNPs was estimated at 20 nm, which is in good agreement with the transmission electron micrograph of the particles. The particle size distribution of AuNPs measured by DLS is presented in Figure 1B. The result of DLS measurement showed a median hydrodynamic diameter (D50) of 21.5 nm, which is in good agreement with the UV–visible absorption spectrum and transmission electron micrograph of the particles.

### Assessment of the Binding of AuNPs to Avidin

**FIGURE 2:**
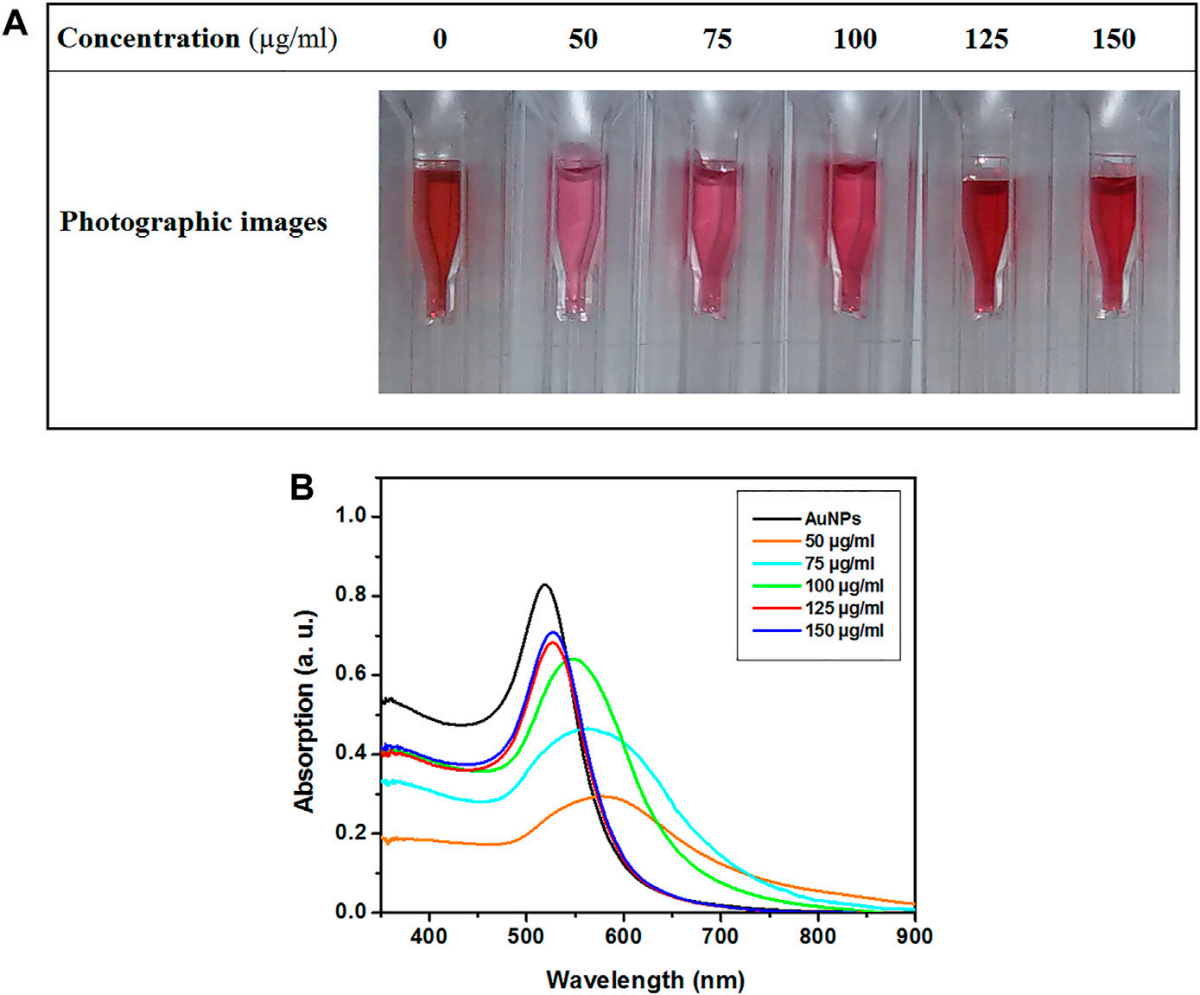
The photographic images **(A)** and UV-Visible absorption spectra **(B)** of avidin–AuNPs after adding NaCl.

The color change in the samples and red-shift in the absorption spectra of samples were the criteria to distinguish the stable samples from unstable ones. Color variation in avidin-AuNPs after adding NaCl is presented in Figure 2A. The color of the samples prepared with avidin at concentrations of 125 and 150 μg/ml remained unchanged. The color of the sample prepared with avidin at concentration of 100 μg/ml showed slight change and the samples prepared with avidin at concentrations of 50 and 75 μg/ml underwent an evident color change. The UV-Visible absorption spectra of avidin–AuNPs following addition of NaCl are presented in Figure 2B. Absorption spectra of the samples prepared with avidin at concentrations of 125 and 150 μg/ml exhibited a smaller red-shift than other samples.

Citrate-reduced colloidal AuNPs have a negative charge, and their stability is of electrostatic type. These nanoparticles are sensitive to the electrolyte concentration. When the concentration of the electrolyte increases, the nanoparticles quickly aggregate, with a color change of the colloid from red to purple or blue. When macromolecules such as proteins cover the surface of AuNPs, polymeric stability is developed. In polymeric stability, the colloidal AuNPs are less sensitive to the rise in the electrolyte concentration. Thus, if the electrolyte concentration increases, no color change can be observed (Chi et al., 2010; Yokota, 2010; Halliwell and Gwenin, 2013; Ríos-Corripio et al., 2013).

The most common method of evaluating the electrostatic binding of AuNPs to protein is Zigmondy’s test. In brief, various concentrations of the protein solution are mixed with samples of the colloidal AuNPs. Then, NaCl solution is added to these samples. The stable sample is the one that does not change color with a minimum concentration of protein. UV–visible absorption spectroscopy can be used for a more in-depth investigation in a way that, the sample which exhibits least red-shift in SPR position is considered the one with best polymeric stability (Slot and Geuze, 2007; Ríos-Corripio et al., 2013). The criteria of selecting the stable conjugates included the sample with a minimal concentration of avidin for developing suitable polymeric stability. Thus, based on the photographic images presented in Figure 2A and the spectra presented in Figure 2B, the sample prepared with avidin at concentration of 125 μg/ml was selected as a stable sample.

### Assessment of the Binding of Avidin-AuNPs to H-1-Biotin

**FIGURE 3:**
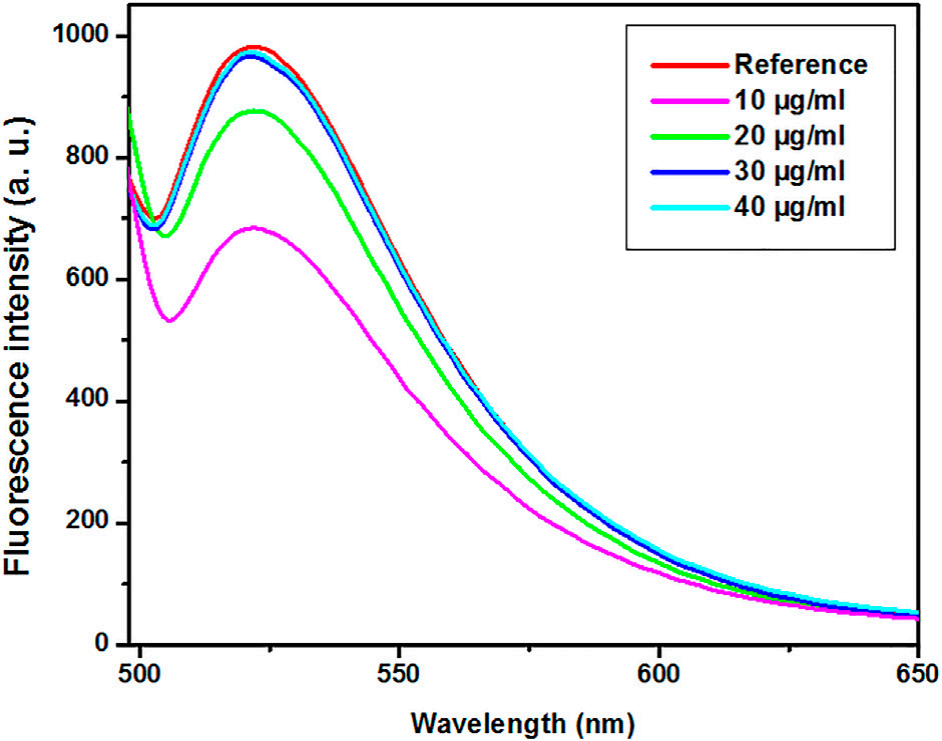
The fluorescence emission spectra of biotin-4-fluorescein after adding various concentrations of H-1-biotin. Reference (Biotin-4-flourescein).

Biotin-4-fluorescein was applied to evaluate the avidin-AuNPs’ binding to the H-1-biotin. When H-1-biotin molecules cover the surface of avidin-AuNPs, biotin-4-fluorescein cannot bind to avidin-AuNPs, and consequently, biotin-4-fluorescein molecules enter the supernatant during centrifugation. The fluorescence emission spectra of biotin-4-fluorescein after adding various concentrations of H-1-biotin are presented in Figure 3. The criteria for selecting the optimized sample included the sample with a minimal concentration of H-1-biotin that has minimal decreased fluorescence intensity at 522 nm. Therefore, based on the spectra presented in Figure 3, the sample prepared with H-1-biotin at concentration of 30 μg/ml was selected as an optimized sample. It is worth mentioning that the biotin-avidin complex is the strongest non-covalent interaction between a ligand and a protein. The bond formation between avidin and biotin is fast, and once formed, is not affected by organic solvents, extremes of pH, and other denaturing agents (Bender, 2003; Liebesa and Marksa, 2014).

### Detection Assay of M1 GAS

**FIGURE 4:**
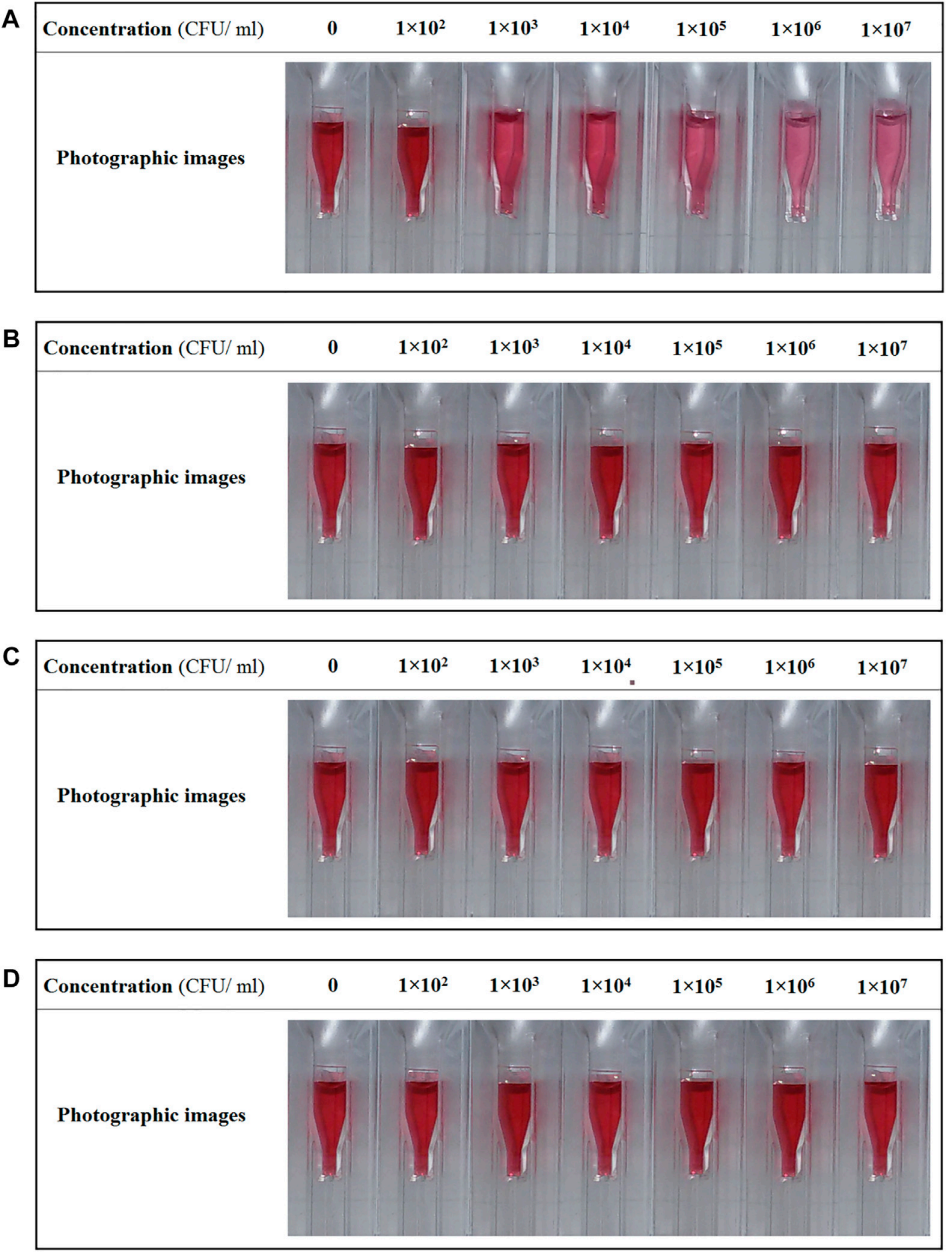
The photographic images of H-1-AuNPs before and after adding saliva samples infected with M1 GAS **(A)**, M6 GAS **(B)**, M12 GAS **(C)**, and *E. coli* O157:H7 **(D)** at concentrations of 1×10^2^, 1×10^3^, 1×10^4^, 1×10^5^, 1×10^6^, and 1 × 10^7^ CFU/ml. Concentration of 0 CFU/ml (as reference, H-1-AuNPs containing uninfected saliva sample). H-1-AuNPs could detect M1 GAS in a concentration range of 1 × 10^3^–1×10^6^ CFU/ml based on color changes.

The no bacterial contamination of saliva sample of a healthy individual with GAS was confirmed by Pouya Gene Azma Company (Confirmation certificate NO: 140011-272). Then, this saliva sample was used as a medium for the detection assay of M1 GAS. The color change in the samples and the value of red-shift in the absorption spectra of samples were the criteria to assess the functionality of H-1-AuNPs. Color variation in H-1-AuNPs before and after adding saliva samples infected with M1 GAS, M6 GAS, M12 GAS, and *E. coli* O157:H7 at concentrations of 1 × 10^2^, 1 × 10^3^, 1 × 10^4^, 1 × 10^5^, 1 × 10^6^, and 1 × 10^7^ CFU/ml is presented in Figure 4. The color of the sample prepared with M1 GAS at concentration of 1 × 10^2^ CFU/ml remained unchanged. The color of the samples prepared with M1 GAS at concentrations of 1 × 10^3^ and 1 × 10^4^ CFU/ml showed slight change and the samples prepared with M1 GAS at concentrations of 1 × 10^5^ and 1 × 10^6^ CFU/ml underwent an evident color change from red to purple. The color of the sample prepared with M1 GAS at concentration of 1 × 10^7^ CFU/ml was similar to the concentration of 1 × 10^6^ CFU/ml (Figure 4A). Furthermore, the color of the samples prepared with M6 GAS, M12 GAS, and *E. coli* O157:H7 at concentrations of 1 × 10^2^, 1 × 10^3^, 1 × 10^4^, 1 × 10^5^, 1 × 10^6^, and 1 × 10^7^ CFU/ml remained unchanged (Figures 4B–D). These results indicated that H-1-AuNPs could specifically and qualitatively detect M1 GAS in a concentration range of 1 × 10^3^–1×10^6^ CFU/ml based on color changes.

**FIGURE 5:**
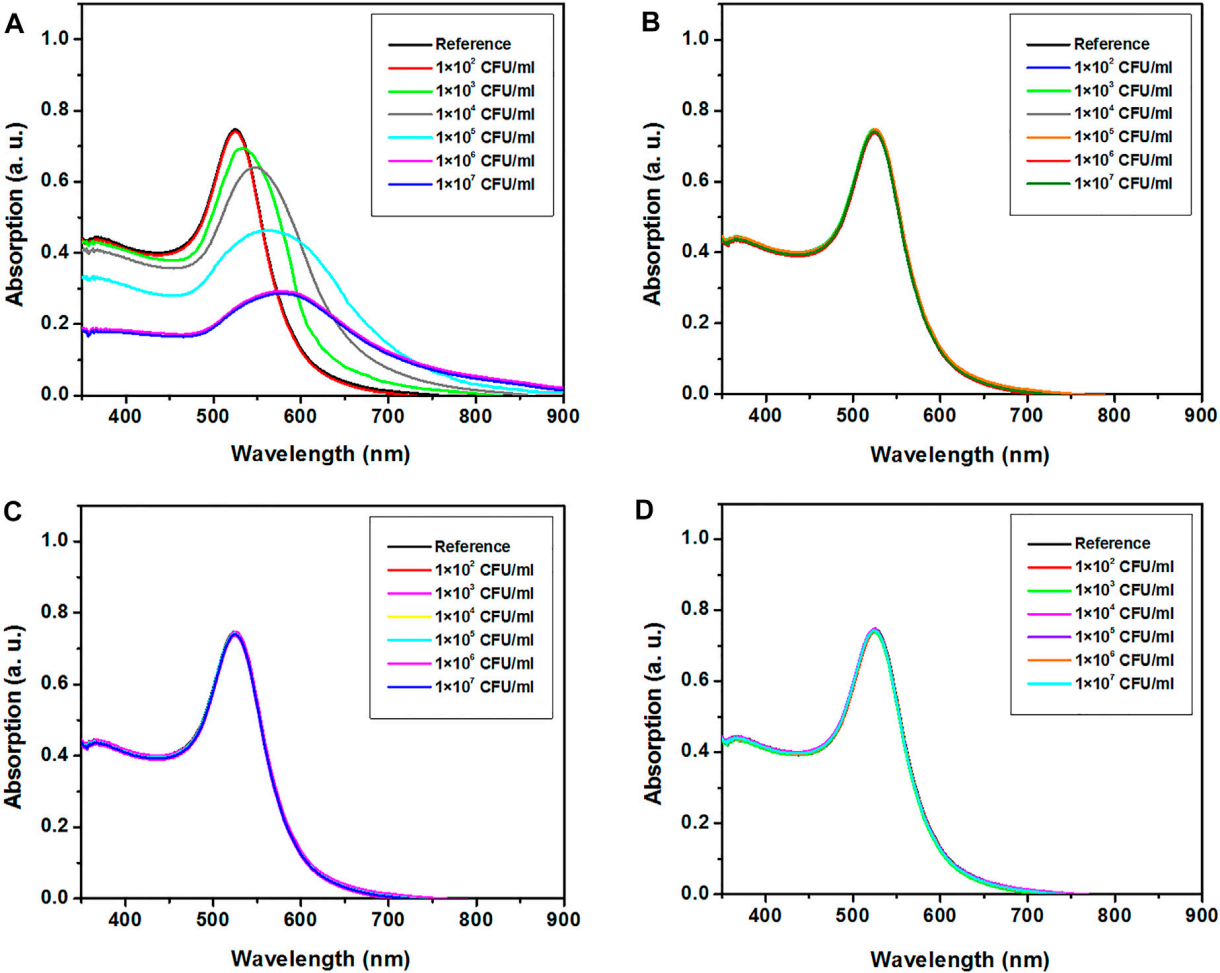
UV–visible absorption spectra of H-1-AuNPs before and after adding saliva samples infected with M1 GAS **(A)**, M6 GAS **(B)**, M12 GAS **(C)**, and *E. coli* O157:H7 **(D)** at concentrations of 1×10^2^, 1×10^3^, 1×10^4^, 1×10^5^, 1×10^6^, and 1 × 10^7^ CFU/ml. Reference (H-1-AuNPs containing uninfected saliva sample). H-1-AuNPs could detect M1 GAS in a concentration range of 1 × 10^3^–1×10^6^ CFU/ml based on the value of red-shift in SPR position.

On the other hand, the UV–Visible absorption spectra of H-1-AuNPs before and after adding saliva samples infected with M1 GAS, M6 GAS, M12 GAS, and *E. coli* O157:H7 at concentrations of 1 × 10^2^, 1 × 10^3^, 1 × 10^4^, 1 × 10^5^, 1 × 10^6^, and 1 × 10^7^ CFU/ml are presented in Figure 5. The criteria for quantitative detecting M1 GAS included the value of red-shift in the absorption peak position. The SPR position of the sample prepared with M1 GAS at concentration of 1 × 10^2^ CFU/ml remained unchanged. The samples prepared with M1 GAS at concentrations of 1 × 10^3^, 1 × 10^4^, 1 × 10^5^, and 1 × 10^6^ CFU/ml showed red-shifts of 14, 27, 41, and 56 nm in SPR position, respectively. The SPR position of the sample prepared with M1 GAS at concentration of 1 × 10^7^ CFU/ml was similar to the concentration of 1 × 10^6^ CFU/ml (Figure 5A). Moreover, The SPR position of the samples prepared with M6 GAS, M12 GAS, and *E. coli* O157:H7 at concentrations of 1 × 10^2^, 1 × 10^3^, 1 × 10^4^, 1 × 10^5^, 1 × 10^6^, and 1 × 10^7^ CFU/ml remained unchanged (Figures 5B–D). These results showed that H-1-AuNPs could specifically and quantitatively detect M1 GAS in a concentration range of 1 × 10^3^–1 × 10^6^ CFU/ml based on the value of red-shift in SPR position.

**FIGURE 6:**
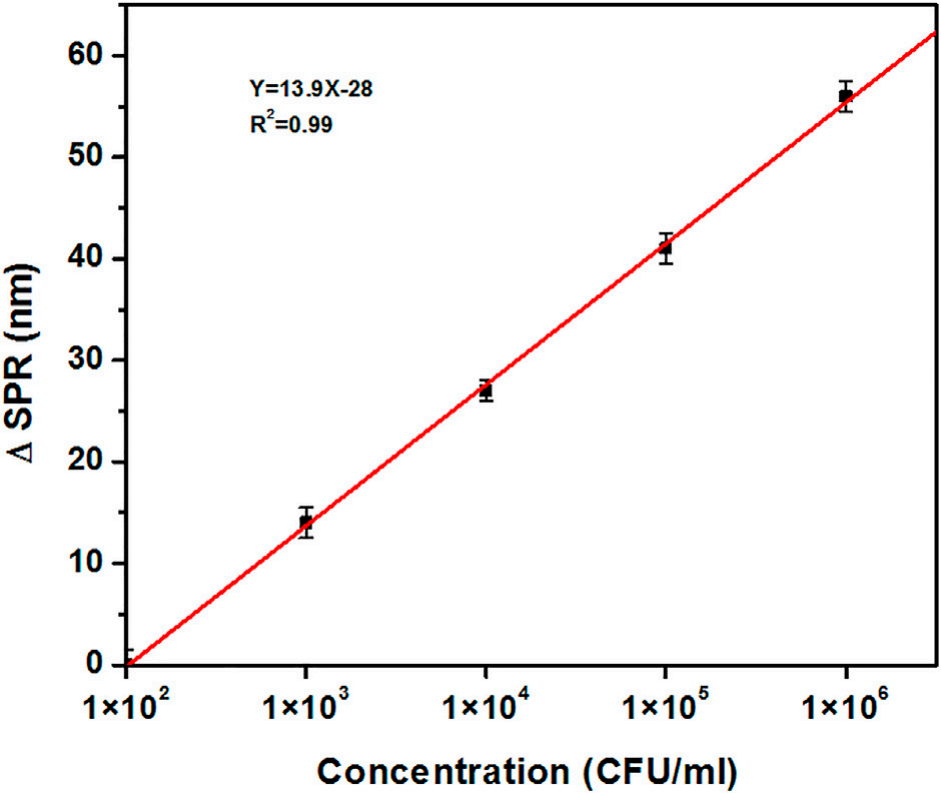
The calibration curve of H-1-AuNPs for concentrations of 1×10^2^, 1×10^3^, 1×10^4^, 1×10^5^, and 1 × 10^6^ CFU/ml of M1 GAS in terms of variations in the red-shift. Calibration curve shows a linear trend.

Based on the value of red-shift in SPR position, a calibration curve for detection of M1 GAS at concentrations of 1 × 10^2^, 1 × 10^3^, 1 × 10^4^, 1 × 10^5^, and 1 × 10^6^ CFU/ml was plotted (Figure 6). It is noteworthy that this calibration curve shows a linear trend, which indicates the good functionality of H-1-AuNPs for the detection of M1 GAS.

The results of the detection assay showed a short detection time (20 min), which is in good agreement with the detection time of previously reported studies (5–10 min), as well as a wide detection range (1 × 10^3^–1 × 10^6^ CFU/ml) in comparison with previously reported studies (9 × 10^3^–1 × 10^5^ CFU/ml) (Quidel Corporation 2015; U.S. Food and Drug Administration 2013; Wang et al., 2010). The comparison of H-1-AuNPs with previously reported biosensors for detection of GAS is presented in Table 1. Although, the commercially available products (US FDA approved) such as Sofia, BD Veritor, and ID NOW can help in the accurate diagnosis of streptococcal pharyngitis (Bulut et al., 2020; Thompson and McMullen, 2020; Quidel Corporation 2015; U.S. Food and Drug Administration 2013; U.S. Food and Drug Administration 2018; Abbott Diagnostics Scarborough 2020), they are not widely used by clinicians worldwide, mainly because of their high price. Besides, the functionality of these products is dependent on the instrument, making them difficult to use. As a result, a novel biosensor for quick diagnosis of streptococcal pharyngitis with cheap manufacturing costs and no reliance on the specialized equipment in qualitative detection is urgently needed. It is worth mentioning that sugar codes usually have relatively low price and high structure stability and on the other hand, nanobiosensors based on aggregation of AuNPs have various benefits like high sensitivity, easy-to-use for qualitative detection without dependence on the specialized instrument, and high speed in analyses. Hence, the development of nanobiosensors that target sugar code-lectin interactions in pathogens might be a promising option for diagnosing infectious disorders such as streptococcal pharyngitis.

**TABLE 1:** Comparison of H-1-AuNPs with previously reported biosensors for detection of GAS.

Bioreceptor	Analysis	Limit of detection	Detection time	Target serotypes	References
Antibody	Qualitative	9 × 10^3^ CFU/ml	5 min	All	Quidel Corporation .(2015)
Antibody	Qualitative	1 × 10^5^ CFU/ml	5–10 min	All	U.S. Food and Drug Administration (2013)
Nucleic acid	Qualitative	Not Reported	6 min	All	Abbott Diagnostics Scarborough (2020)
Carbohydrate	Qualitative and quantitative	1.5 × 10^2^ CFU/ml	20 min	M1	This work

Due to wide linear detection range (1 × 10^3^–1 × 10^6^ CFU/ml), short detection time (20 min), good reproducibility, easy-to-use for qualitative detection without dependence on the specialized instrument, and relatively low production cost, H-1-AuNPs can be regarded as an affordable nanobiosensor for the detection of M1 GAS that may lead to a strategic breakthrough in the diagnosis of streptococcal pharyngitis. However, detection assay using the standard strain of M1 GAS was one of the main limitations of our study. For this reason, it will be important in future studies to perform detection assay using clinical strains of M1 GAS before the clinical application of H-1-AuNPs. Another limitation of the present study was that H-1-AuNPs could detect only M1 GAS (the most common cause of streptococcal pharyngitis), but not all serotypes. Owing to great benefits (especially high sensitivity and easy-to-use for qualitative detection without dependence on the specialized instrument), design of nanobiosensor based on the aggregation of AuNPs to detect other important serotypes of GAS, such as M6 GAS and M12 GAS can be considered in future studies.

## Conclusion

**FIGURE 7:**
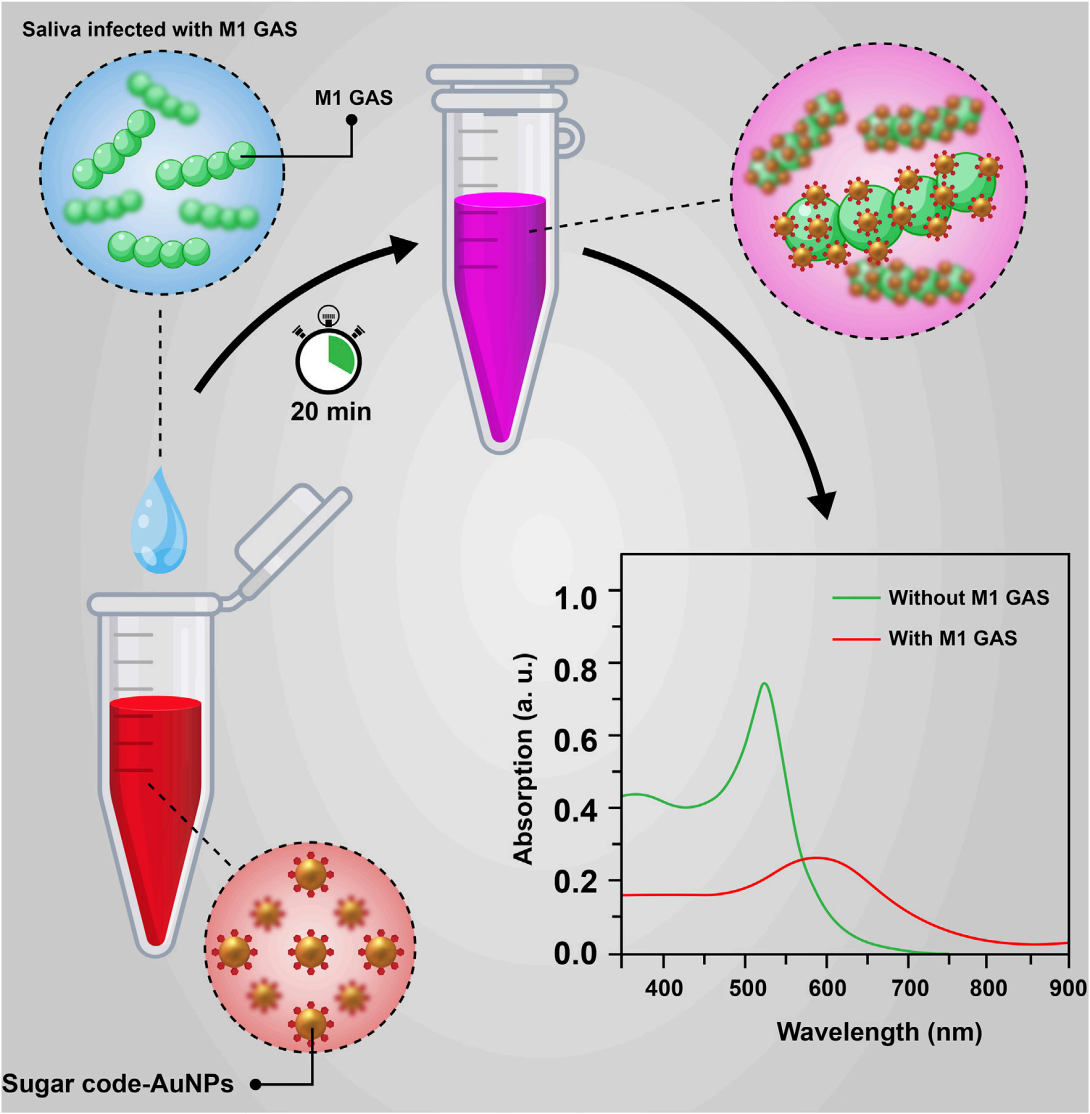
A schematic illustration of nanobiosensor based on the aggregation of sugar code-AuNPs for the rapid detection of M1 GAS.

In summary, we have successfully developed a relatively affordable nanobiosensor based on the aggregation of H-1-AuNPs for the rapid, qualitative, and quantitative detection of M1 GAS (Figure 7). The results of the detection assay represented a wide detection range (1 × 10^3^–1 × 10^6^ CFU/ml) with linear response, indicating the good functionality of this nanobiosensor for the specific detection of M1 GAS. The other attractive features of this nanobiosensor include a short detection time of 20 min, good reproducibility, easy-to-use for qualitative detection without dependence on the specialized instrument, and relatively low production cost. Consequently, this work can be considered a key to opening new doors in the rapid diagnosis of streptococcal pharyngitis in future studies.

## Data Availability

The original contributions presented in the study are included in the article/Supplementary Material, further inquiries can be directed to the corresponding author.
